# Recent Progress on the Characterization of Cellulose Nanomaterials by Nanoscale Infrared Spectroscopy

**DOI:** 10.3390/nano11051353

**Published:** 2021-05-20

**Authors:** Qianqian Zhu, Rui Zhou, Jun Liu, Jianzhong Sun, Qianqian Wang

**Affiliations:** 1 Biofuels Institute, School of the Environment and Safety Engineering, Jiangsu University, Zhenjiang 212013, China; happyzqq@ujs.edu.cn (Q.Z.); zr2172773442@163.com (R.Z.); junliu115142@ujs.edu.cn (J.L.); 2 Key Laboratory of Biomass Energy and Material, Institute of Chemical Industry of Forest Products, Chinese Academy of Forestry, Nanjing 210042, China; 3 State Key Laboratory of Bio-based Materials and Green Papermaking, Qilu University of Technology, Jinan 250353, China; 4 State Key Laboratory of New Textile Materials and Advanced Processing Technologies, Wuhan Textile University, Wuhan 430200, China

**Keywords:** cellulose nanomaterials, cellulose nanocomposite, nanoscale resolution

## Abstract

Researches of cellulose nanomaterials have seen nearly exponential growth over the past several decades for versatile applications. The characterization of nanostructural arrangement and local chemical distribution is critical to understand their role when developing cellulose materials. However, with the development of current characterization methods, the simultaneous morphological and chemical characterization of cellulose materials at nanoscale resolution is still challenging. Two fundamentally different nanoscale infrared spectroscopic techniques, namely atomic force microscope based infrared spectroscopy (AFM-IR) and infrared scattering scanning near field optical microscopy (IR s-SNOM), have been established by the integration of AFM with IR spectroscopy to realize nanoscale spatially resolved imaging for both morphological and chemical information. This review aims to summarize and highlight the recent developments in the applications of current state-of-the-art nanoscale IR spectroscopy and imaging to cellulose materials. It briefly outlines the basic principles of AFM-IR and IR s-SNOM, as well as their advantages and limitations to characterize cellulose materials. The uses of AFM-IR and IR s-SNOM for the understanding and development of cellulose materials, including cellulose nanomaterials, cellulose nanocomposites, and plant cell walls, are extensively summarized and discussed. The prospects of future developments in cellulose materials characterization are provided in the final part.

## 1. Introduction

Cellulose materials are renewable resources available in large quantities [1,2,3]. An in-depth understanding of the properties of cellulose materials will promote the development of renewable biomaterials and bioenergy, thereby enhancing the sustainability of our society [4,5]. In this review, cellulose materials are intended to contain a broader range of materials, including cellulose nanomaterials (CNMs), cellulose nanocomposites, and plant cell walls. CNMs are new classes of materials consisting of cellulose particles with at least one dimension smaller than 100 nm [6,7,8,9,10,11]. CNMs are known for their renewability, biocompatibility, biodegradability, low cytotoxicity, and excellent mechanical performances [12]. CNMs nanocomposites find many applications as packing materials, biomedical materials, smart materials, energy storage materials, etc. [13]. Due to all these advantages and potential applications, research on cellulose materials is under widespread scrutiny and investigation in both academic and industrial communities [14,15]. The excellent and unique properties of cellulose nanocomposites are determined by the distinct allocation and structural arrangement of cellulose particles within the composites, and also other factors such as the inherent properties of cellulose particles and matrix, and their concentration and interface. Understanding wood cell wall architecture provides vital information for improving the properties of wood composites [16]. Therefore, the consistency, reliability, accuracy, and spatial resolution of characterization in chemical distribution and structural arrangement are critical for increasing the mechanistic understanding of cellulose materials.

Best practices and techniques for cellulose materials characterization, such as CNMs, particularly in particle morphology, surface chemistry, mechanical properties, and toxicity, are extensively summarized and reviewed, which represents a solid foundation for CNMs characterization for both academic pursuits and industrial practice [6]. A collection of standardized protocols was also established or recommended. Cellulose material properties, such as particle morphology (scanning electron microscopy, SEM; transmission electron microscopy, TEM; atomic force microscopy, AFM), elemental composition (X-ray photoelectron spectroscopy, XPS; energy-dispersive X-ray spectroscopy, EDS; inductively coupled plasma mass spectrometry, ICP-MS), surface modification (Fourier transform infrared spectroscopy, FTIR; nuclear magnetic resonance, NMR), suspension properties (ultraviolet-visible spectroscopy, UV-Vis; zeta potential, rheology), solid-state properties (X-ray powder diffraction, XRD; Raman spectroscopy), and mechanical strength (dynamic mechanical analysis, DMA; tensile testing) were characterized by the aforementioned techniques in brackets. The need to characterize cellulose materials has driven significant advances in characterization capabilities.

The well-established tools, such as AFM and FTIR, were widely used for cellulose materials characterization. As a powerful and multifunctional technique, AFM offers a multitude of different acquisition modes and provides valuable information at the nanoscale on CNMs morphology [6,17], orientation [18,19], architecture deconstruction [20,21], mechanical strength [22], and adhesive properties [23]. FTIR spectroscopy is a bulk chemical structure analysis technique, which links IR absorptions with specific kinds of bonds, functional groups, and composite components. The area or volume-averaged spectral data cannot give regiospecific information. The powerful micro-FTIR spectroscopy enables the chemical mapping across the studied samples at the micron level [16,24,25,26,27]. The spatial resolution of micro FTIR spectroscopic imaging is determined by the diffraction limit associated with the mid-IR light at wavelengths of 2.5–10 μm. With the development of synchrotron-source IR spectroscopy and other approaches in recent years, it is now possible to provide an increase of the signal and better signal-to-noise ratio. However, the best achieved spatial resolution was still roughly limited to several micrometers. In brief, AFM with nanometer resolution can provide the morphology of cellulose materials without chemical information, whereas micro-FTIR provides chemical information with limited spatial resolution. 

Although significant progress has been made with the aforementioned techniques for cellulose materials characterization, there are still many practical challenges that need to be efficiently and economically solved [4,28,29]. For example, the characterization of the interaction between CNMs and water molecules presents a practical challenge in dewatering, rehydration, and redispersing because of the strong tendency to form hydrogen bonding. Because of the nanoscale dimension, high surface area, and low percolation thresholds, the characterization of CNMs dispersibility in different media and their distribution in the composite matrix is challenging [6,10]. The interfaces (sharp two-dimensional boundaries between two phases) and interphases (transition zones between two phases in a composite) play a vital role in the mechanical properties of the resulting cellulose nanocomposites. Thus, understanding the nature of the interface/interphase is important when developing cellulose nanocomposites. Characterizing the interface and interphase of cellulose nanocomposites with morphological and chemical information simultaneously at nanoscale for a more fundamental understanding is a real challenge due to their small size and complex interactions, and also the lack of suitable tools. To meet these challenges, nanoscale IR spectroscopy including atomic force microscope based infrared spectroscopy (AFM-IR) and infrared scattering scanning near field optical microscopy (IR s-SNOM) have been adopted to realize nanoscale spatially resolved imaging for both morphological and chemical information by the integration of AFM with IR spectroscopy [30,31]. Nanoscale IR spectroscopy provides information-rich IR spectra that allow for nanoscale chemical identification of different groups and compositions based on standard FTIR libraries. The state-of-the-art AFM-IR and IR s-SNOM techniques can reach 10-nm spatial resolutions.

This review first describes the urgent need for consistent, reliable, and accurate characterization techniques with simultaneously morphologic and chemical information at the nanoscale for cellulose materials. As a potential solution, nanoscale IR spectroscopies, including AFM-IR and IR s-SNOM, are introduced in Section 2. Section 3 presents the state-of-the-art research applications where nanoscale IR spectroscopy leads as a novel multi-scale visualization and characterization technique for both morphological and chemical information of cellulose materials. Specific applications, including CNMs, CNMs nanocomposites, and plant cell walls, are emphasized. Section 4 summarizes the capabilities and limitations of nanoscale IR spectroscopy. Lastly, the prospects on the research direction by nanoscale IR spectroscopy for cellulose materials characterization are provided in Section 5.

## 2. Overview of AFM-IR and IR s-SNOM

**Figure 1:**
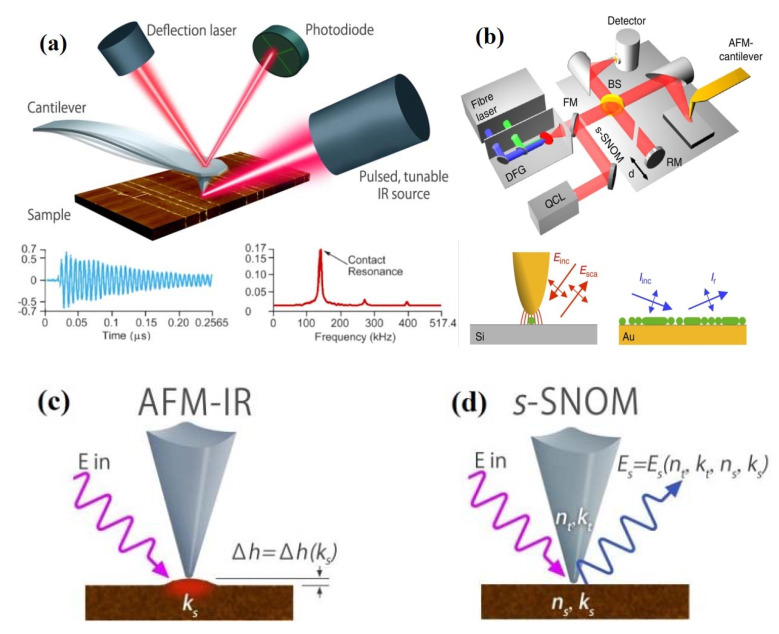
Schematic diagram of AFM-IR (**a**), IR s-SNOM (**b**), and their working principles (**c**,**d**). Reproduced with permission from [37,43]. Copyright Macmillan Publishers Limited, 2013; Copyright Microscopy Society of America; Copyright Anasys Instruments (Now Bruker Corporation), 2015.

Nanoscale infrared spectroscopies of both AFM-IR and IR s-SNOM are hybrid technologies that integrate the spatial resolution of AFM and the chemical analysis capability of IR spectroscopy [32]. With those nanoscale infrared spectroscopies, it is possible to determine and image local chemical composition below the diffraction limit. AFM-IR measures the photothermal-induced resonance effect by measuring either the local thermal expansion or temperature rise with AFM tip after IR absorption by a sample [33,34]. The thermal expansion or temperature change is proportional to the infrared light absorption coefficient of the sample. Thus, the detected signal can be converted into the vibrational spectrum of the sample. The IR s-SNOM technique, by contrast, determines the amount of scattered light from the specimen [35,36]. The light scattered was influenced by the comprehensive optical properties of the AFM probe, specimen, and underlying substrate. The schematic diagrams of AFM-IR, IR s-SNOM, and their differences in principles are shown in Figure 1. AFM-IR equipment consists of a pulsed tunable IR source with an AFM (Figure 1a,c). When the pulsed IR light is absorbed by the sample near the AFM tip, a rapid photothermal expansion of the sample occurs creating a transient cantilever oscillation, which can be further converted to a local IR absorption spectrum as a function of wavenumber [33]. The sharp AFM tip and infrared spectroscopy endow AFM-IR with the chemical analysis and imaging capabilities at the nanoscale. A pulsed tunable IR source with an AFM is also an essential accessory for IR s-SNOM (Figure 1b,d). On the other hand, IR s-SNOM collects the scattered near-field of light from a metallic AFM tip with the sample underneath [35]. The wavelength or wavenumber-dependent optical and chemical properties can be obtained via the collected light with amplitude and phase properties [36,37]. More basic principles on the theory for AFM-IR and IR s-SNOM techniques are available in [34,38,39,40,41,42].

As such, AFM-IR performs best on samples with a larger thermal expansion (the tendency of matter to change its volume and shape in response to temperature variation), whereas IR s-SNOM is the best technique for materials with a larger light scattering coefficient (the ability of matter to scatter photons). Both complementary modes can be acquired by combining AFM-IR and IR s-SNOM into a single instrument. This was achieved in nanoIR2-s^TM^ (Anasys Instruments, Santa Barbara, CA, USA), neaSNOM system (Neaspec GmbH, Munich-Haar, Germany), and later versions by the pioneered companies of Anasys Instruments (Now part of Bruker Corporation) and Neaspec GmbH (Now Part of Attocube Systems), respectively.

## 3. AFM-IR and IR s-SNOM Application in Cellulose Materials

Advances of AFM-IR and IR s-SNOM technology make it possible to perform IR spectroscopic mapping and chemical analysis at the nanoscale with spatial resolution beyond the Abbe diffraction limit for the characterization of nanomaterials and nanostructures. AFM-IR is now recognized as one of the most important novel techniques for analyzing various systems, such as polymer blends, organic fibers, composites, multilayer thin films in addition to biological samples [33,34,44], while IR s-SNOM has demonstrated great popularity in studying monolayered and inorganic samples as well as soft materials [35,38,45].

In addition to its capability of achieving nanoscale chemical analysis and composition mapping, several practical benefits of AFM-IR and IR s-SNOM are listed below: (i) Simple sample preparation. Solution deposition, spin coating, and microtome are the most adopted processes for flat sample preparation. Top-down and top-side illumination endow a substantially broader range of samples with arbitrary thickness to be examined on arbitrary substrates [46]. (ii) High-speed and non-destructive measurement. Spectra can be obtained in seconds or less. (iii) Insensitivity to fluorescence and no need for staining steps. This is very useful, especially for characterizing the plant cell wall with lignin component. (iv) Rich, interpretable nanoscale infrared spectra. Nanoscale IR spectroscopy and imaging offer a wide range of morphological and chemical properties of the specimens at the nanoscale [31,40]. A preliminary comparison of the advantages and disadvantages of AFM-IR and IR s-SNOM is briefly summarized in Table 1.

Since its first demonstration to characterize cellulose materials, the application of nanoscale IR spectroscopy and imaging to study the properties of cellulose materials are receiving great attention from the research community. The progressive increase in the number of publications in recent years reflects the rising interest and importance of such new techniques for cellulose materials characterization. This paper aims to provide a thorough literature review on the characterization of cellulose materials using AFM-IR and IR s-SNOM. Table 2 summarizes the major applications of nanoscale IR spectroscopy techniques for cellulose materials.

### 3.1. AFM-IR and IR s-SNOM Application in CNMs Characterization

**Figure 2:**
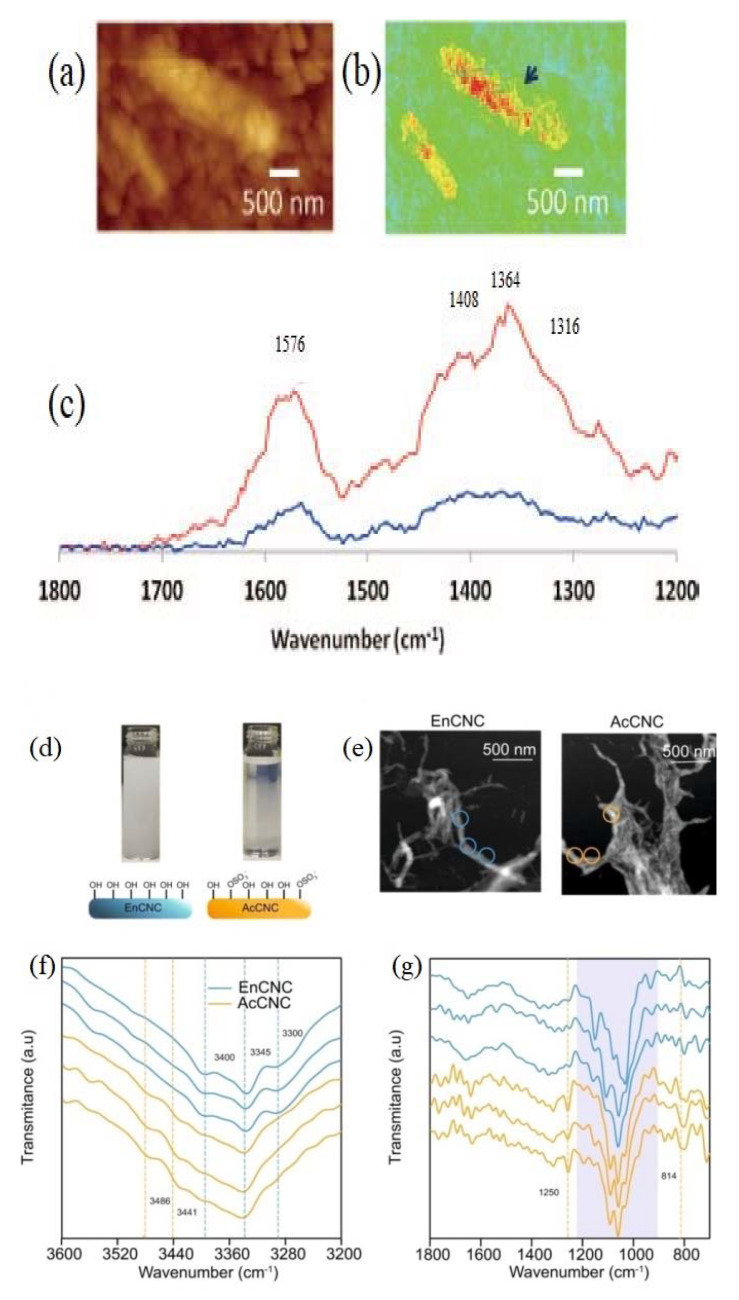
(**a**) AFM topography image of MFC in cellulose matrix, (**b**) NanoIR image at 1360 cm^−1^, (**c**) NanoIR spectra; (**d**) CNCs suspension produced by enzymatic and acid hydrolysis, (**e**) Topography images from EnCNC and AcCNC, (**f**) NanoIR spectra for EnCNC and AcCNC nanocrystals in 3600 to 3200 cm^−1^ (**f**) and 1800 to 750 cm^−1^ (**g**). Reproduced with permission from [46]. Copyright Cambridge University Press, 2012. Reproduced from [47].

Elucidation of the structural organization of CMMs and chemical modified cellulose materials is crucial to understand their role in different applications. The nanoscale IR spectroscopy provides structure arrangement and chemical composition information from nanoscale domains and structural organization of CNMs. The pioneering AFM-IR experiments that characterize microfibrillated cellulose (MFC) as a paper additive were conducted by Marcott et al. [46]. MFC is short rod-like cellulose fibrils with high crystallinity. As the first example for cellulose materials characterized by AFM-IR, the AFM topography image (Figure 2a) and IR absorbance image (Figure 2b) acquired with the light source tuned to 1360 cm^−1^ have been demonstrated as nanoscale chemical spectroscopy with a spatial resolution of 50 nm. The high crystalline domains of MFC in the paper were visualized.

Nanoscale IR spectroscopy can effectively characterize the distribution of functional groups of cellulose nanomaterials and cellulose derivatives at the nanoscale, such as sulfate half-ester groups and aromatic groups [47,48]. The structurally and chemically different CNC particles obtained from sulfuric acid hydrolysis (AcCNC) and enzymatic hydrolysis (EnCNC) were examined with a neaSNOM system using Au-coated AFM tips as shown in Figure 2d–g. The peaks at 814 cm^−1^ and 1250 cm^−1^ attributed to sulfate groups indicated the esterification of surface hydroxy groups of cellulose. Styrene grafting, chloroacetylation, amination, and protonation of MCC were also verified by tuning the IR source at the characteristic wavelengths of 1160, 1490, and 1735 cm^−1^. The AFM-IR result demonstrated that grafting and copolymerization of styrene occurred mainly on the cellulose surface.

**Figure 3:**
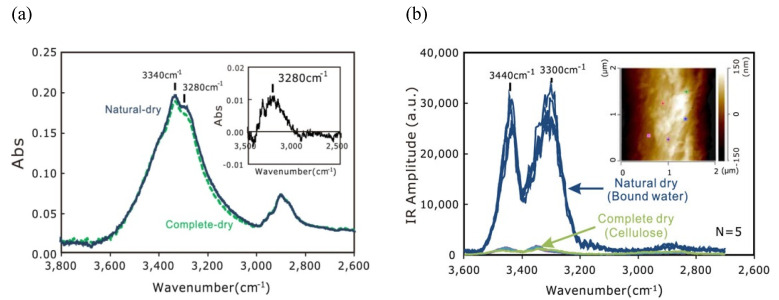
(**a**) Conventional ATR-IR spectra of dried cotton cellulose; (**b**) Direct observation of the interaction between water and cellulose by AFM-IR. Reproduced with permission from [49]. Copyright American Chemical Society, 2020.

As compared to bulk IR spectroscopy, nanoscale IR spectroscopy has great potential for an in-depth analysis of the mechanism of interactions between CNMs and small molecules, such as water [49]. Figure 3a shows slight differences of the OH-stretch band in the 3800–2600 cm^−1^ region in the conventional ART-IR spectra between different dried cotton cellulose. Due to the low resolution and high noise level, bulk ART-IR cannot provide genuine microscopic information on the bound water localized at the outermost surface of differently dried cotton cellulose. This limitation of bulk ART-IR was overcome by AFM-IR. The difference in the coefficient of thermal expansion between water and cellulose made the AFM-IR measurement possible. As a surface-sensitive approach, AFM-IR indicated how and where the bound water exists on the cellulose. The state of hydrogen bonding in bound water is different from that in bulk water. The bulk water has a broad trapezoidal shape spectrum, whereas the bound water on the cellulose surface exhibits two decoupled stretching modes of OH groups, as shown in Figure 3b, which originate from the effects of the hydrophobic air-water interface (at lower wavenumber side) and hydrophilic water–cellulose interface (at higher wavenumber side), respectively. 

### 3.2. AFM-IR and IR s-SNOM Application in CNMs Nanocomposites Characterization

**Figure 4:**
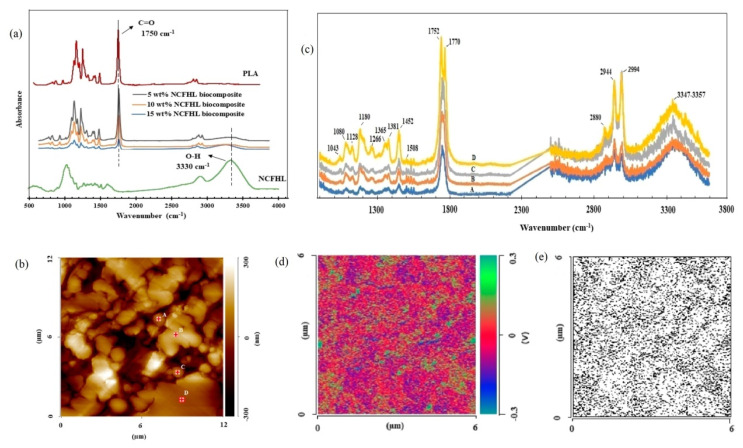
(**a**) Bulk FTIR spectra for nanocellulose fibrils, neat PLA, and their nanocomposites (**b**) AFM topography image of the nanocomposites (**c**). Corresponding NanoIR spectra obtained for areas in (**b**). (**d**) NanoIR (Anasys Instruments, Santa Barbara, CA, USA) images with the IR laser at 3330 cm^−1^ (nanocellulose fibrils characteristic peak), and its corresponding binary image (**e**). Reproduced with permission from [51]. Copyright American Chemical Society, 2018.

CNMs nanocomposites are becoming increasingly important in the academic and industrial community, where CNMs with enhanced properties are being added to bulk polymers to achieve improved properties and performances [43]. Well dispersed cellulose nanomaterials can enhance the properties of cellulose nanocomposites. Nonetheless, little is understood about the exact mechanism and extent of interfacial chemistry in the interphase regions. This requires a localized probe to fully understand the local structure and chemistry, interphase formation, interfacial chemistry, and mechanical properties of CNMs nanocomposites. To achieve a deep understanding of the micro/nano arrangements and structures of CNCs, the distribution of CNCs in polyurethane foam nanocomposites was verified by IR s-SNOM imaging and nano-FTIR spectroscopy with a neaSNOM system [50]. Microdomains of strong IR phase contrast at 1050 cm^−1^ indicated the even distribution of CNCs in the nanocomposites. Similar research was conducted to study the distribution of nanocellulose fibrils with high lignin content (NCFHL) in the PLA matrix and their interface properties [51]. Ultrathin cellulose nanocomposite crosssection was used for AFM-IR imaging on the silicon substrate. NanoIR spectra complement well with the conventional FTIR spectra as shown in Figure 4. Slight differences in peak position and intensity were detected, which may be more local characteristics reflected by nanoIR spectra. The intense IR absorption indicates the presence of higher concentrations of nanocellulose fibrils (Figure 4c). Nanoscaled IR maps were converted into binary images to examine the interphase distribution. The quantitative analysis of the binary images indicated that approximately 22% of the total area was filled with NCFHL fibrils, while neat PLA occupied about 32% as a separate phase. Approximately 46% region in the binary map was attributed to the transition phase or interfacial area. This large proportion of interfacial area within the specimen was ascribed to the reaction between highly dispersed NCFHL and PLA molecules, and also lignin as a strong compatibilizer during the pressing of the biocomposites at high temperature. 

Local and regional distribution of CNF and starch distribution was correlated to the topographic information with the local chemical analysis [54,55]. A new peak at a wavelength of 1630 cm^−1^ in the local IR spectrum indicated the formation of hydrogen bonding between cellulose and starch. The dispersion behavior of adipic acid molecules modified CNCs in polybutylene adipate-co-terephthalate (PBAT) was examined by AFM-IR at 1700 cm^−1^ for C=O absorption [53]. The good dispersion and interaction of adipic acid-modified CNCs in PBAT were attributed to the hydrophilic core and hydrophobic shell structure of modified CNCs. 

Tip functionalization could be utilized to improve the spatial resolution of nanoscale IR spectroscopy [52]. The refractive index contrast of CNMs was greatly enhanced by using a polydimethylsiloxane (PDMS) functionalized tip. The fine structure of CNCs alignment inside the PAN-CNCs fiber can be demonstrated. The strands of polyacrylonitrile-cellulose nanocrystals (PAN-CNCs) nanofibers show strong thermal expansion at 1462 cm^−1^, whereas the bare CNCs debris exhibits weak thermal expansion. In contrast, the bare CNCs particles illustrate a strong thermal expansion at 1027 cm^−1^, while the CNCs inside the PAN fiber display weak signals. The refractive indices for PAN and CNCs are comparable to each other. The refractive index contrast between CNCs and PAN was greatly enhanced with the assistance of a polydimethylsiloxane (PDMS) functionalized tip. With the enhanced refractive index contrast, the substructure of PAN-CNCs nanofiber and bare CNCs particles were simultaneously visualized. The CNCs alignment within the PAN-CNCs nanofibers was clearly manifested. Other functionalized tips such as Pt or Au coated AFM tips were also used to enhance the signals [47,50,51].

### 3.3. AFM-IR and IR s-SNOM Application in CNMs Nanocomposites Characterization

It is of great interest to characterize the nanostructure and local arrangement of plant cell wall components [21]. The local properties of plant cell walls were strongly correlated with the nanoscale architecture of its components. The morphological and chemical structure of wood cell walls at the nanoscale resolution or even molecular level could promote a deep understanding of fine structures of plant cell walls after pretreatment and modification. AFM-IR provides a new method to investigate the chemistry of plant cell walls at the nanoscale resolution without employing special treatments.

**Figure 5:**
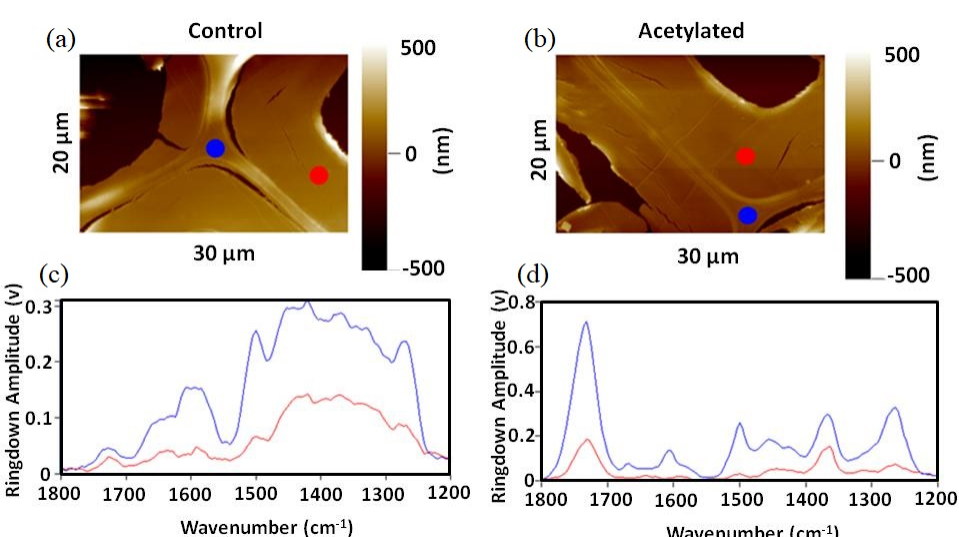
AFM topography images of (**a**) a control wood sample and (**b**) an acetylated wood sample, and their corresponding nanoIR spectra (**c**,**d**) at indicated locations. Reproduced with permission from [56]. Copyright Anasys Instruments (Now Bruker Corporation), 2012.

To our best knowledge, the first study using nanoscale IR spectroscopy to characterize the wood cell wall was reported by Marcott et al. using an AFM-IR instrument at spatial resolutions on the order of 100 nm with a tunable IR laser source that illuminates the sample from below [56]. The underneath laser illumination requires that the wood samples must be thin enough. The optimal thickness is less than 1 μm (500 nm, in this study). IR spectra as a function of spatial position for both transverse and longitudinal cross-sections of the untreated and acetylated wood samples were examined by AFM-IR, as shown in Figure 5. Lignin content in compound corner middle lamella as reflected by the band near 1500 cm^−1^ was higher than that in the S2-layer [56]. The acetylation intensified the carbonyl band at 1736 cm^−1^. The broadband IR spectrum obtained at any arbitrary point provides insight into microdomain composition and leads to an increased understanding of the cell wall structure and properties of biomaterials. Updated versions of AFM-IR instruments with top-down or top-side illumination do not have such limitations in that specimens of arbitrary thickness can be examined on arbitrary substrates. Similarly, the distribution of functional groups in native and chemical modified wood was examined with an excellent spatial resolution of 16 nm [60].

NanoIR imaging has also been employed to capture the removal of lignin occurring upon pretreatments of the plant cell wall [58]. Local IR spectra of the pretreated sample exhibited a significant drop in lignin content, confirming alterations in topography. The correlation of the AFM morphological image, IR-sensitive images, the mechanical phase image enabled a deep understanding of mechanical properties together with the chemical components and structure of cell walls [64]. The nanoscale compositional variations in plant cell walls characterized by nanoscale IR spectroscopy were also used to interpret plant cell wall physiological phenomena, such as heartwood formation and water transport in the xylem [59,65]. The spectral variations at 1660 cm^−1^ and 1640 cm^−1^ in heartwood and sapwood were identified. The intensity of the peak at 1660 cm^−1^, which was assigned to the ethylenic C=C and C=O bond stretches of lignin, decreased from sapwood to heartwood, whereas the intensity of the peak at 1660 cm^−1^ attributed to a carbonyl stretching and hydrogen bonding to the carbonyl group increased. The possible explanation for this variation is that phenolic precursors of extractives accumulate in the sapwood, are oxidized and condensed in the transition zone, and diffuses into the heartwood. Distributions of hydrophobic compounds and compounds with a high electric potential of pit membranes in water-conducting cells may influence the water transport system in plants. Further research is required to illustrate the effects of plant species and water availability variations.

The accurate evaluation of the interphase properties is critical to understanding how the polymer interacts with the wood cell wall components and optimizing the design and fabrication of the wood-plastic composites (WPCs). The specific molecular-scale penetration and interactions between phenol-formaldehyde (PF) resin and wood cell wall were in situ identified by AFM-IR for the first time [62]. The intensity of the characteristic peaks for cell wall and PF resin gradually changes across the distance from the resin region, resin-cell wall interphase area, to cell wall area as shown in the AFM-IR images. Similarly, the penetration of isocyanates (pMDI) in the wood cell wall was also evaluated by AFM-IR [63]. The molecular-scale penetration and interactions between pMDI and cell wall strengthened the connections between the cell wall and polymer and thus improved the mechanical strength.

### 3.4. AFM-IR and IR s-SNOM Application for Other Cellulose-Containing or Cellulose-Derived Materials

**Figure 6:**
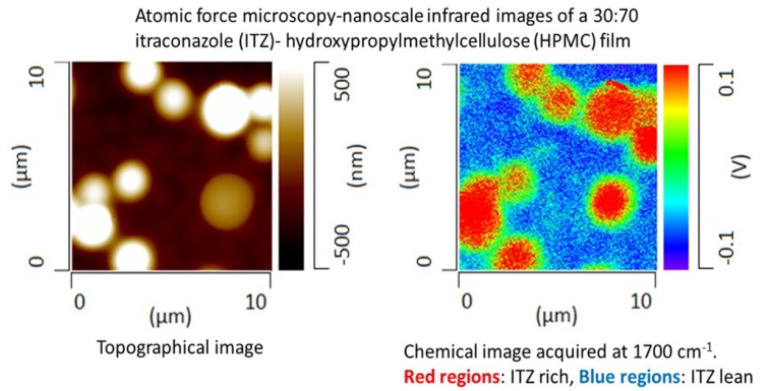
The miscibility of itraconazole-hydroxypropyl methylcellulose blends (30:70) characterized by AFM-IR. Reproduced with permission from reference [66]. Copyright American Chemical Society, 2015.

It was challenging to characterize the drug-polymer miscibility due to the similarity in the glass transition temperature and small size domain. Recently, AFM-IR has been applied to evaluate the miscibility of drug and hydroxypropyl methylcellulose as shown in Figure 6 [66,67]. The AFM-IR data showed that the phase separation in a drug (Itraconazole, Telaprevir) and hydroxypropyl methylcellulose dispersion occurred at the submicron scale. These results provide new insights into the interphase/interface behavior of drug–polymer dispersions.

Cellulose fiber was used as a precursor fiber for carbon fiber. The evolution of functional groups (C–O and C=O) and the changes in micro-domain structure during the synthesis of cellulose-based carbon fibers were examined by nanoscale infrared spectroscopy [68]. The IR spectra of C–O and C=O indicated that the skin and core of carbon fibers were not pyrolyzed homogenously. AFM-IR techniques have also been used to detect the polypyrrole wearable nanofilm devices on the surface of cellulose-based substrates [69].

## 4. Challenge and Limitation of Nanoscale IR Spectroscopy in Cellulose Materials Characterization

Although offering remarkable advantages, nanoscale IR spectroscopy and imaging also have some limitations in cellulose materials characterization [33,34]. The resolution of nanoscale IR spectroscopy is often limited by the properties of cellulose specimens, such as thickness, roughness, and thermal diffusivity. What’s more, these influences are wavelength related leading to more complex changes in relative signal intensities. Better resolution of experimental data may result in poor reproducibility. This poor reproducibility may not only be due to the differences in sample preparation but also closely related to the chemical inhomogeneity of the cellulose materials. This is especially true for plant cell walls. Thickness-induced chemical shifts of IR s-SNOM resulted in chemical and structural analysis more complex than those for conventional bulk spectra. The versatility of nanoscale IR spectroscopy and imaging is at the cost of the ability to directly correlate the obtained spectra information to the quantitative characterization of chemical and structural properties [70,71]. Complex data processing methods with certain degrees of signal degradation still cannot eliminate background interferences. A huge challenge for AFM-IR is the difficulty to quantify the relationship between the AFM-IR tip-sample contact dynamics and signal intensity, which depends on the stability of the AFM operation and the sample local thermomechanical properties [72]. To date, the ability of AFM-IR as an analytical tool to characterize the single cellulose molecule and single chemical bond scale has remained elusive over a wide range of cellulose materials. The limited resolution was due to a relatively large volume excited by IR lasers in the sample. The application of AFM-IR was limited to relatively flat and large self-assembled aggregates or monolayers composed of several hundreds of molecules. Many spatial resolutions during nanoscale IR measurements are not determined. This may be due to the difficulty in determining the spatial resolution of thick cellulose samples with irregular geometry and unclear margin. In general, IR spectroscopy is sensitive to water molecular and atmospheric gases. This is also true for nanoscale IR spectroscopy. Cellulose, as a hydrophilic and hygroscopic material, is also sensitive to water. There is no doubt that humidity will have a great influence on the characterization of cellulose materials by Nanoscale IR spectroscopy with such high resolution. Unfortunately, the effects of humidity on the nanoscale IR spectroscopy measurements of cellulose materials were not systematically studied. In some measurements, humidity data were not even provided. Recently, nanoscale IR spectroscopy instruments with humidity and temperature control accessories are available, which may be able to eliminate the interference of humidity and temperature on the characterization of cellulose materials.

## 5. Summary and Outlook

Nanoscale IR spectroscopy provides insightful data on the chemical structure of cellulose materials with nanoscale spatial resolution. The most recent advances of AFM-IR and IR s-SNOM on the characterization of CNMs, cellulose nanocomposites, and plant cell walls were extensively explored. Progress is still underway, and nanoscale IR spectroscopy now offers an exciting future for the application of cellulose materials. AFM-IR and IR s-SNOM have been used to identify and quantify polymer components in blends, characterize interfaces/interphase in composites, assess the local crystallization, and even reverse engineer multilayer films. Additionally, morphological and chemical maps could be further processed to get rich information. These techniques and protocols can also be applied to characterize cellulose materials. More than techniques for compositional analysis and chemical mapping at the nanoscale spatial resolution as discussed above, AFM-IR and IR s-SNOM can also obtain mechanical, thermal, and electrical property mapping at employed wavelength or spectra at employed positions for cellulose nanocomposites. Nevertheless, there are still many other opportunities to further anticipated developments. With the rapid theoretical and technical improvement in nanoscale IR spectroscopy, it is expected that the characterization of cellulose materials by AFM-IR and IR s-SNOM will increase significantly in the coming decades. In the foreseeable future, a more mechanistic understanding of CNMs composites will be obtained and more powerful CNMs nanocomposites will be developed.

## Figures and Tables

**Table 1:** Advantages and disadvantages of AFM-IR and IR s-SNOM techniques.

Technique	Advantages	Disadvantages
**AFM-IR**	Non-destructive, ambient conditions Fine spatial resolution morphological and chemical imaging Suitable for organics, such as polymer, blends, and composites as well as biological samples Point IR spectra acquisition Directly correlates to FTIR	Background interference Tip contamination Signal enhancement by gold-coated probes and substrates Reduced peak intensity as compared to bulk IR spectra Resolution and signal intensity is limited by sample properties (thickness, smoothness thermal diffusivity, etc.)
**IR s-SNOM**	Non-destructive, ambient conditions Fine spatial resolution Optical properties including amplitude and phase Suitable for inorganics, such as photonics and 2D materials that efficiently scatter light	Background interference Tip contamination Reduced peak intensity as compared to bulk IR spectra Need theoretical models to interpret data Artifacts such as band distortion and thermal drift

**Table 2:** Application of AFM-IR and IR s-SNOM for the characterization of cellulose materials.

Sample	Sample Preparation	Instrument	AFM Tip	Spatial Resolution	Spectral Range /cm^−1^	Research Area/Topic	Reference
Cellulose Nanomaterials							
Microfibrilated cellulose (MFC)	Solution deposition; spin coating; microtomed	NanoIR	NA.	50 nm	1200–1800	Local crystallinity analyses	[46]
Cellulose Nanocrystals (CNCs)	Single CNC particles	neaSNOM	Au-coated tips	nanoscale	800–3600	Cellulose polymorphy and sulfur analyses	[47]
Microcrystalline cellulose (MCC)	Solution deposition	NanoIR	NA.	NA.	1160, 1490, 1735	Distribution of functional groups	[48]
Single cotton cellulose fiber	Single fibers	NanoIR2	NA.	10 nm	2600–3800	Observation of water on cellulose surfaces	[49]
**CNMs Nanocomposites**							
CNCs/polyurethane (PU) foams	foam cross sections	neaSNOM system	Pt-Si or Au coated tips	a few ten nanometers	900–1400	CNCs distribution in PF foam	[50]
Nanocellulose fibrils with high lignin content (NCFHL)/polylactic acid (PLA) composites	Microtomed; several nanometers	NanoIR2	Au coated silicon nitride tip	tens of nanometers	800–4000	Dispersion of various phases and interfacial regions	[51]
CNCs/Polyacrylonitrile (PAN) nanofiber	Nanofiber from electrospinning	VistaScope microscope + quantum cascade lasers	NCH-Au 300 kHz noncontact tip	nanoscale resolution	800–1800	Heterogeneous substructure of PAN/CNCs nanofiber	[52]
CNCs/ poly(butylene adipate-co-terephthalate) (PBAT) nanocomposites	Suspension deposition	NanoIR2-s	AFM tip	NA.	1600–1800	Dispersion of CNCs and modified CNCs in PBAT	[53]
Starch/cellulose nanofibers (CNF) nanocomposites	Film casting on gold-coated silicon substrate	NanoIR2-s	AFM tip	10 nm	1530–1845	Topography correlated with local chemical analyses	[54,55]
**Plant Cell Wall**							
Wood cell wall	Microtomed; 500 nm	NanoIR	AFM tip	100 nm	1200–1800	Microdomains spectroscopic characterization	[56]
Wood cell wall	Microtomed; 5 μm	NanoIR2	Au-coated tip	nanoscale resolution	1530–1810	Chemical alterations and inhomogeneity of cell wall	[57,58]
Wood cell wall	Microtomed; 10 μm	NanoIR	AFM tip	NA.	1200–1800	Heartwood formation process	[59]
Wood cell wall	Microtomed; smooth surfaces	Homemade nanoscale IR	Pt-coated silicon probe	16 nm	900–1800	Nanoscale chemical features of wood substrates	[60]
Wood cell wall	Microtomed	NanoIR2	NA.	sub-100 nm	1550–1800	Local chemical changes and lignin rearrangement	[61]
Wood cell wall /polymer composites	Microtomed; 200 nm	NanoIR2	Au-coated silicon nitride tip	100 nm	900–1800	Molecular-scale interactions of polymer and cell wall	[62,63]

NA.: Not Available. The information is not provided in the cited reference.

## Data Availability

Not applicable.
